# The Influence of Negative Affect and Food Stimuli on Cognitive Flexibility in Adolescents With Anorexia Nervosa

**DOI:** 10.1002/erv.3159

**Published:** 2024-11-28

**Authors:** Meital Gil, Yael Latzer, Noa Tziperman, Dan Farbstein, Helene Sher, Noam Weinbach

**Affiliations:** ^1^ School of Psychological Sciences University of Haifa Haifa Israel; ^2^ Faculty of Social Welfare and Health Sciences University of Haifa Haifa Israel; ^3^ Eating Disorders Institution Psychiatric Division Rambam Health Care Campus Haifa Israel; ^4^ Pediatric Psychosomatic Department Safra Children's Hospital Sheba Medical Center Tel Hashomer Israel; ^5^ Department of Psychiatry Soroka Medical Center Beer Sheva Israel

**Keywords:** adolescents, anorexia nervosa, cognitive flexibility, negative affect, task switching

## Abstract

**Objective:**

Inflexible thinking among individuals with anorexia nervosa (AN) was proposed to reflect difficulties in set‐shifting. However, studies assessing set‐shifting in AN often find mixed results, especially in adolescent samples. It has been proposed that affective states and exposure to disorder‐salient stimuli may modulate executive functions in AN. The current study examined the influence of induced negative emotion on the ability to shift toward or away from a food categorisation task among adolescents with AN.

**Methods:**

The study included 47 adolescents with AN and 41 healthy adolescents who performed a modified task‐switching paradigm.

**Results:**

No indication of general set‐shifting difficulties among adolescents with AN was found. Nevertheless, the results showed that when negative emotion was induced, adolescents with AN shifted from a non‐food categorisation task to a food categorisation task with greater efficiency compared to a neutral emotion condition. Emotion and switch type did not influence set‐shifting abilities among healthy adolescents.

**Conclusion:**

The findings indicate automatic and more efficient switching towards preoccupation with food among adolescents with AN while experiencing negative emotion. The results emphasise the important role played by situational factors in modulating cognitive abilities in individuals with AN.

**Summary**

- The study assessed how induced negative emotion influences shifting away and towards a food categorisation task among adolescents with anorexia nervosa (AN).
- The results revealed that when negative emotion was induced, adolescents with AN shifted towards a food categorisation task from a non‐food categorisation task more efficiently.
- The results of the study highlight the important role played by affective states and disorder‐salient stimuli in modulating executive functions among individuals with AN.

## 1 Introduction

Anorexia nervosa is a serious eating disorder characterised by restricted food intake, fear of gaining weight, and a disturbance in the way the body is experienced (American Psychiatric Association 2013). Anorexia nervosa commonly onsets during adolescence. Treatments for AN have limited success with remission occurring only in 30%–40% of the patients (Fichter et al. 2017). Research endeavours attempt to map potential factors that may hinder successful treatment in AN. Inflexible and rigid thinking style is a common characteristic among individuals with AN that has been suggested to constitute a barrier for changing pathological eating behaviours and cognitions during psychotherapy (Crane, Roberts, and Treasure 2007; Treasure and Schmidt 2013). Thus, it is important to gain insight into the mechanisms that may subserve rigid thinking among individuals with AN.

Set shifting is an executive function that is theorized to underlie cognitive flexibility (Smith et al. 2018). Set shifting refers to the ability to switch between multiple tasks, operations, or mental sets (Monsell 1996). Previous studies have suggested that inefficient set‐shifting contributes to cognitive rigidity among individuals with AN (Keegan, Tchanturia, and Wade 2021; Roberts et al. 2007; Tchanturia et al. 2011), and may serve as a trait marker of AN (Holliday et al. 2005; Keegan, Tchanturia, and Wade 2021; King et al. 2019). Nevertheless, empirical evidence regarding set‐shifting difficulties in AN is inconsistent, especially in studies on adolescents with AN (Herbrich et al. 2018; K. Lang et al. 2014; Miles et al. 2020; Westwood et al. 2016). These inconsistencies may suggest that set‐shifting difficulties are not a trait marker of AN but result from enduring or acute illness. However, studies have not shown that set‐shifting abilities among adolescents with AN differ as a function of illness stage (see review in Miles et al. 2020). As such, a more in‐depth investigation into the nature of set‐shifting abilities among adolescents with AN is required.

Set shifting is a dynamic process that operates in different environmental settings and conditions. Thus, it is reasonable to theorise that set‐shifting abilities may interact with situational factors that contribute to eating disorder symptoms. State negative affect, for example, is a significant risk factor for the future development of AN as has been demonstrated in several prospective studies (Stice et al. 2021 2022). Moreover, difficulty regulating negative affect is a well‐established transdiagnostic factor across all types of eating disorders (Leppanen et al. 2022; Mallorquí‐Bagué et al. 2018). Additionally, momentary experience of negative affect among individuals with AN precedes restrictive eating episodes (Engel et al. 2016). As such, the experience of state negative affect may exacerbate eating disorder symptoms. Negative affect was also shown to modulate various executive functions, including set‐shifting (Hsieh and Lin 2019). Hence, it is likely that an aberrant pattern of set‐shifting in individuals with AN may be more salient during the experience of negative emotion. No previous study has assessed the potential role of negative affect in modulating set‐shifting abilities among individuals with AN.

Another situational factor that may influence cognitive functioning in individuals with AN is exposure to disorder‐related stimuli (Simon, Stopyra, and Friederich 2019). In particular, exposure to food stimuli was found to modulate various executive functions among patients with eating disorders. For example, abnormal patterns of inhibitory control in adolescents with AN were demonstrated only when food stimuli were embedded within an inhibitory control task (Weinbach, Bohon, and Lock 2019). Furthermore, exposure to food stimuli results in a strong attentional bias among individuals with AN compared to healthy individuals (Werthmann et al. 2019). To the best of our knowledge, the influence of food exposure on set‐shifting abilities among adolescents with AN has not been examined. Given that adolescents with AN are highly preoccupied with the selection and categorisation of different types of food (Lloyd and Steinglass 2018), tasks that require shifting towards or away from a food categorisation task may expose aberrant patterns of set‐shifting among these individuals.

A common method for assessing set‐shifting in experimental settings is using the task switching paradigm (Braver, Reynolds, and Donaldson 2003). In this task, participants perform two classification tasks that require attention to different features of a given target stimulus. Cues that appear before the target inform participants which task they should carry out when the target appears. In “repeat trials”, a cue signalling the same task repeats in trial N and trial N‐1. “Switch trials” involve consecutive trials in which a different task cue appears in trial N and trial N‐1. Participants are commonly slower and less accurate in “switch trials” compared to “repeat trials”. This effect is termed the “switching cost” and serves as a marker of set‐shifting abilities (Braver, Reynolds, and Donaldson 2003).

Previous studies have used the task‐switching paradigm to show set‐shifting difficulties among adults with AN compared to healthy controls (Berner et al. 2019; Dann et al. 2023; King et al. 2019). However, these studies did not manipulate participants' affective states and did not require engaging in tasks that involved food stimuli. Thus, it is difficult to infer from these studies how set‐shifting interacts with situational factors that are associated with eating disorder symptoms. Furthermore, these studies focused on adult patients with AN, who may be more susceptible to cognitive decline due to prolonged illness duration (Fonville et al. 2014). To date, no study assessed set‐shifting using the task‐switching paradigm in a sample of adolescents with AN.

The goal of the current study was to assess the influence of negative affect on the ability to shift toward and away from a food categorisation task among adolescents with AN and healthy controls. For this purpose, we developed an Emotion/Food Task Switching paradigm. This paradigm involved alternating between a food sorting task (i.e., food task) and a colour sorting task (i.e., non‐food task). Participants were required to repeat the same task or switch between the two tasks on various trials. In this way, the task allowed assessing the ability to mentally switch from a food task to a non‐food task and from a non‐food to a food task separately. Additionally, in separate blocks, negative or neutral images were embedded between trials. This allowed assessing the switching processes in negative or neutral emotional contexts. We hypothesised that adolescents with AN would show greater difficulty switching from a food task to a non‐food task compared to switching from a non‐food towards a food task. This effect was expected to reflect difficulty disengaging from a food task. Moreover, it was expected that this effect would be more robust in negative compared to neutral emotion blocks. No differences in switching abilities as a function of task type and emotion block were expected among healthy adolescents.

## 2 Methods

### 2.1 Participants

The study included 50 adolescents with AN and 44 healthy adolescents (see power analysis below). All of the participants were females in the age range of 12–18 years. Data from three adolescents with AN and three healthy adolescents were excluded from the analysis due to low accuracy in the Emotion/Food Task‐Switching paradigm (above 2.5 standard deviations from the mean). As such, the final analyses included 47 adolescents with AN and 41 healthy adolescents. All adolescents with AN were receiving inpatient care but were at different levels of treatment. Most patients (*n* = 44) were not underweight while participating in the study (i.e., Expected Body Weight > 85%). However, despite the weight gain, all patients initially received a diagnosis of full AN. Clinical and demographic data of the participants appear in Table 1. A power analysis using G*Power (Faul et al. 2007) showed that for a power of > 80% with an a priori alpha set at *p* = 0.05 and an expected medium‐sized effect of η^2^_*p*_ = 0.06, 39 participants are required for detecting within‐between subject variables interactions.

**TABLE 1:** Clinical and demographic measures of the participants.

	Adolescents with AN (*n* = 47)	Healthy adolescents (*n* = 41)	*p*‐value	Cohen's d
Age (years)	15.4 (1.5)	16.6 (1.5)	< 0.001	0.27
Illness duration (months)	25.12 (13.49)	—	—	—
A report of previous hospitalizations (%)	31.9%	—	—	—
Use of psychotropic medications (%)	68%	0%	—	—
Lowest BMI	16.77 (2.14)	—	—	—
%EBW	96.42 (11.98)	104.10 (17.44)	0.02	−0.51
Wechsler IQ scaled score ‐ Vocabulary	11.14 (3.23)	11.70 (3.50)	0.44	−0.17
Wechsler IQ scaled score ‐ Matrices	10.27 (2.50)	10.61 (3.32)	0.59	−0.11
EDE‐Q‐Global score	4.38 (0.99)	1.04 (1.07)	< 0.001	3.25
DASS – depression	13.08 (4.48)	3.22 (3.94)	< 0.001	2.33
DASS – anxiety	11.02 (5.06)	2.41 (3.57)	< 0.001	1.94
DASS – stress	13.59 (4.14)	4.02 (4.49)	< 0.001	2.22
CFS	3.41 (0.75)	4.56 (0.64)	< 0.001	−1.62
Comorbid diagnoses (%)				
Major depressive disorder/dysthymia	40.42%	2.43%		
Anxiety disorder	36.17%	2.43%		
Obsessive‐compulsive disorder	19.14%	2.43%		
Post‐traumatic stress disorder	8.51%	0%		
Attention deficit hyperactivity disorder	2.08%	4.87%		

*Note:* Standard deviations appear in parentheses.

Abbreviations: BMI, body mass index; CFS, Cognitive Flexibility Scale (lower scores represent less flexibility); DASS, Depression Anxiety Stress Scales (higher scores indicate greater severity); %EBW, % expected body weight; EDE‐Q, Eating Disorder Examination Questionnaire (higher scores mean greater symptoms' severity).

### 2.2 Recruitment

Adolescents with AN were recruited from two eating disorders inpatient centres (“Soroka” and “Rambam” medical centres) between the years 2020–2023. Healthy adolescents were recruited via advertisements on social networks (i.e., Facebook). All participants had to be within the age range of 12–18 years. Participants in the patient group had to have a diagnosis of AN based on the Diagnostic and Statistical Manual of Mental Disorders, fifth edition (DSM‐5; American Psychiatric Association 2013). Exclusion criteria for both groups were the presence of any neurological illness, brain injury, or trauma that could interfere with neurocognitive functioning, as assessed via self‐report.

### 2.3 Clinical Assessment

Diagnosis of AN and comorbid disorders (see Table 1) were assessed using the Mini‐Kid International Neuropsychiatric Interview (Sheehan et al. 2010). Among those with AN, 37 were diagnosed with a restrictive subtype and 9 with a binge eating/purging subtype of AN.

### 2.4 Measures

#### 2.4.1 Expected Body Weight (%EBW)

Height and weight were measured on the day of the assessment using a weight scale and a stadiometer. Expected body weight (%EBW) was then calculated based on the 50th percentile for height, age, and gender from the Centers for Disease Control and Prevention (CDC) charts.

#### 2.4.2 Intelligence Assessment

Potential differences in IQ between the groups were assessed using the vocabulary and matrices subsets of the Wechsler Adult Intelligence Scale (WAIS; for participants aged 16 or above; Wechsler 1997) and Wechsler Intelligence Scale for Children (WISC; for participants under the age of 16; Wechsler 2004), with raw scores transformed to scaled scores based on participants' age.

#### 2.4.3 Self‐Report Questionnaires

Eating disorders symptom severity was assessed using the Eating Disorder Examination Questionnaire (EDE‐Q; Fairburn and Beglin 1994). Symptoms of depression, anxiety, and stress (DASS) were assessed using the Depression Anxiety Stress Scales (DASS; Lovibond and Lovibond 1995), and self‐reported cognitive flexibility was assessed using the Cognitive Flexibility Scale (CFS; Martin and Rubin 1995). Additional questionnaires were used as well but are not within the scope of the current research.

#### 2.4.4 Emotion/Food Task‐Switching

**FIGURE 1:**
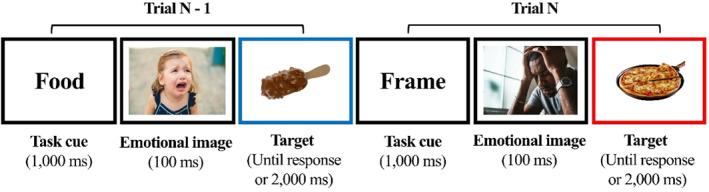
An example of two consecutive trials in a negative emotion block in the emotion‐food task switching paradigm. This example represents a ‘switch from a food’ trial because participants switched from a food task (trial N‐1) to a frame task (trial N).

In each trial of the task, a target picture of high‐calorie food was presented inside a coloured frame (the timing of the events is depicted in Figure 1). Participants were presented with two separate tasks. In the ‘food task’, participants were requested to categorise the taste of the presented food stimuli (e.g., pressing ‘z’ if the flavour was sweet, pressing ‘m’ if the flavour was salty). In the ‘frame task’, they categorised the frame's colour by pressing ‘z’ if the frame around the food picture was coloured in red, and ‘m’ if it was blue (the key choices for each category were counterbalanced across participants). A cue appeared before the target informing participants which task to perform. Specifically, the word ‘Frame’ informed participants to respond according to the frame colour, and the word ‘Food’ informed them to respond according to the food's flavour. Two consecutive trials in which the same task was repeated were coded as ‘repeat‐food task’ or ‘repeat‐frame task’ trials. Two consecutive trials that required alternating between two different tasks were categorised as ‘switch’ trials which were further separated into ‘switch from food’ (i.e., switching from a food task to a frame task) or ‘switch towards food’ (i.e., switching from a frame to a food task) trials. The task included 4 separate blocks (64 trials in each block). Within each block, the proportion of the switch and repeat trials was predetermined to allow a similar amount of repeat and switch trials within a single block. Thus, the trials were presented in a pseudo‐random order.

To manipulate emotional state, the task was separated into a negative or neutral emotion blocks. Specifically, in neutral emotion blocks, an emotionally neutral image appeared after the cue and before the target in each trial. In negative emotion blocks, an emotionally negative image appeared (stimuli detailed below). The task included two negative and two neutral emotion blocks. The order of these blocks was counterbalanced across participants. Overall, the task included 256 trials over 4 blocks. Prior to the task, participants engaged in a short practice phase to familiarise themselves with the task and corresponding key presses. This practice included 10 consecutive trials in which participants performed the ‘frame task’. A second practice block included 10 trials of the ‘food task’ (the order of these two practice blocks was counterbalanced), and a third practice block of 20 trials that included alternations between the ‘frame’ and ‘food’ tasks. The practice phase included feedback in case of an erroneous response.

Switching costs were calculated by subtracting response times (RTs) and accuracy rates between switch and repeat trials. The task allowed assessing two types of switching costs. First, switching cost from food (i.e., the cognitive cost of switching away from a food task to a frame task) was calculated as the difference in mean RT and accuracy in ‘switch from food’ trials to that of ‘repeat frame’ trials (i.e., assessing performance in the frame task that was preceded by a food task to performance in a frame task that was preceded by a frame task). Second, switching cost towards food (i.e., the cognitive cost of switching away from a frame to a food task) was calculated as the mean RT and accuracy differences in ‘switch towards food’ trials to ‘repeat food’ trials (i.e., performance in a food task that was preceded by a frame task to performance in a food task that was preceded by a food task). Moreover, the task allowed assessing switching costs away and towards food separately in negative and neutral emotional blocks.

#### 2.4.5 Emotion Pictures

64 Negative and 64 neutral images were selected from the International Affective Picture System (IAPS; P. J. Lang et al. 1997). Neutral images were chosen for their medium valence and arousal ratings based on normative data (*M*_valence_ = 5.53, SD = 1.38; *M*_arousal_ = 3.61, SD = 1.96). Negative images were chosen based on their low valence (i.e., indicating greater negativity ratings) and high arousal (*M*_valence_ = 3.1, SD = 1.63; *M*_arousal_ = 5.32, SD = 2.14). The study only included negative images that were appropriate for adolescents to view. The neutral and negative images had an equal proportion of human, animal, and object pictures. Each image was presented twice throughout the task.

#### 2.4.6 Food Pictures

High‐calorie food images were selected from a food picture database (Blechert et al. 2014) with an equal proportion of sweet and salty foods. In the current study, 32 images of sweet food, and 32 images of salty food were chosen. Each food image was presented four times throughout the task.

### 2.5 Procedure

The study is part of a larger research project that assessed executive functions in adolescents with AN. Ethical approvals have been obtained from the University of Haifa IRB committee (479/20) and Helsinki committee at each of the two recruitment sites (“Soroka”: 0366‐20‐SOR, “Rambam”: 0837‐20‐RMB). Upon the identification of eligible participants, a research coordinator placed at each site contacted the patient and her parents to obtain informed consent. After the consent process, an online link with the self‐report questionnaires was sent to the adolescent.

Following that, a research coordinator scheduled a meeting with the adolescent either at her home (some meetings were conducted via Zoom due to COVID‐19) or at the local eating disorder unit. During this meeting, participants underwent the IQ assessment, the Mini‐Kid (some patients underwent the Mini‐Kid prior or after this session within the eating disorder unit), and another executive function task that is not within the scope of the current study. Another session was scheduled 7–14 days after the first session and involved the completion of the Emotion‐Food task switching paradigm. Adolescents with AN performed the task within the inpatient unit between supervised meals and thus were under a state of satiety. We did not control for hunger level among healthy adolescents.

### 2.6 Statistical Analysis

Independent *t*‐tests were used to assess potential differences between the groups on demographic and clinical measures (see Table 1). To test the primary hypothesis, two three‐way mixed model ANOVAs were carried out with group (AN/healthy adolescents) as a between‐subject independent variable, switching cost type (towards food/away from food), and emotion block (negative/neutral) as within‐subject independent variables. In the first ANOVA, the dependent measure was switching costs in accuracy and the second ANOVA used switching costs in RTs. Only RTs for correct responses were used in the RT analysis (removing 8.42% of the data). Correct RTs lower than 200 ms were discarded as well as RTs larger or lower than 3 SDs above the mean for each participant in each experimental condition (3.09% of the trials).

Finally, exploratory correlational analyses assessed associations between general switching costs in RTs and accuracy and clinical measures including eating disorder severity (EDEQ‐global score and subscales), self‐reported cognitive flexibility (assessed using the CFS), illness duration, and %EBW.

## 3 Results

Response times and percentage of errors as a function of emotion block, trial type, and group are displayed in Table 2. Raw data have been made publicly available at OSF: https://osf.io/h6wuy/?view_only=a61adaa2488648ed91e7687c358df981.

**TABLE 2:** Response times (RTs) (in ms) and percentage of errors as a function of emotion block, trial type and group.

Trial type	Negative emotion block		Neutral emotion block	
	Adolescents with AN	Healthy adolescents	Adolescents with AN	Healthy adolescents
Repeat food task	732 (5.87%)	763 (5.78%)	729 (6.52%)	744 (5.92%)
Repeat frame task	673 (7.79%)	717 (6.06%)	665 (6.44%)	659 (4.93%)
Switch away from food task	753 (12.58%)	781 (12.23%)	736 (11.41%)	747 (11.48%)
Switch towards food task	759 (7.76%)	797 (9.16%)	778 (11.05%)	816 (10.20%)

Abbreviation: AN, anorexia nervosa.

### 3.1 Clinical and Demographic Measures

Clinical and demographic variables are presented in Table 1. Adolescents with AN had lower %EBW, higher EDE‐Global scores, higher scores of DASS, lower CFS scores, and were younger in age compared to healthy adolescents. No significant difference was found between the groups in IQ scores.

### 3.2 Switching Costs in Error Rates

The ANOVA revealed a significant main effect for switch type *F*(1,86) = 5.94, *p* = 0.017, η^2^_p_ = 0.065, demonstrating that in general, participants were less accurate switching away from a food task compared to switching towards a food task (mean switching costs are displayed in Table 3). There were no main effects for emotion block, *F*(1,86) = 2.21, *p* = 0.141 η^2^_p_ = 0.025, or group, *F*(1,86) = 1.31, *p* = 0.255 η^2^_p_ = 0.015. The three‐way interaction between emotion block, switch type, and group was not significant, *F*(1,86) = 0.44, *p* = 0.509, η^2^_p_ = 0.005. However, we carried out planned comparisons to assess our a‐priori hypotheses. The results revealed that adolescents with AN showed greater accuracy when switching towards a food task compared to switching away from a food task in the negative emotion block, *F*(1,86) = 4.15, *p* = 0.047, η^2^_p_ = 0.046, but not in the neutral emotion block, *F*(1,86) = 0.10, *p* = 0.75, η^2^_p_ = 0.001. Additional analyses were carried out to assess if the greater switching cost demonstrated when switching away from food compared to switching towards food among adolescents with AN in the negative emotion block represents greater difficulty disengaging from a food task, or alternatively, better ability to switch towards a food task. Specifically, we compared each type of switching cost separately between the emotional blocks. The analysis revealed a smaller switching cost towards a food task in the negative compared to the neutral emotion block, *F*(1,86) = 4.85, *p* = 0.033, η^2^_p_ = 0.053, while there was no significant difference in switching costs away from a food task between the two emotion block conditions, *F*(1,86) = 0.02, *p* = 0.896, η^2^_p_ = 0.0001. These results suggest that adolescents with AN had better ability to switch towards a food task when experiencing negative emotion, rather than greater difficulty switching away from a food task.

**TABLE 3:** Switching costs as indicated by response times (RTs) (in ms) and percentage of errors as a function of emotion block, switch type, and group.

Emotion block	Switching cost from food		Switching cost towards food	
	Adolescents with AN	Healthy adolescents	Adolescents with AN	Healthy adolescents
Negative	81 (4.78%)	65 (6.16%)	27 (1.89%)	34 (3.38%)
Neutral	71 (4.97%)	88 (6.54%)	49 (4.53%)	72 (4.28%)

Abbreviation: AN, anorexia nervosa.

Among healthy adolescents, no significant differences in switching cost types were found in the neutral, *F*(1,86) = 1.79, *p* = 0.188, η^2^_p_ = 0.020, nor in the negative emotional blocks, *F*(1,86) = 2.06, *p* = 0.159, η^2^_p_ = 0.023.

### 3.3 Switching Costs in Response Times

The ANOVA revealed a significant main effect for switch type *F*(1,86) = 9.17, *p* = 0.003, η^2^_p_ = 0.096, demonstrating a larger switching cost in RTs when switching away from a food task compared to switching towards a food task. The main effect for emotion block was near the significance level, *F*(1,86) = 3.92, *p* = 0.051, η^2^_p_ = 0.044, demonstrating a smaller switching cost in the negative compared to the neutral emotional block (see Table 3). There was no main effect for group, *F*(1,86) = 0.37, *p* = 0.546 η^2^_p_ = 0.004. The three‐way interaction between emotion block, switch type, and group was not significant, *F*(1,86) = 0.22, *p* = 0.639, η^2^_p_ = 0.003. However, planned comparisons were used to further assess the hypotheses. In accordance with the hypothesis and replicating the pattern demonstrated in the error rates analysis, adolescents with AN displayed a greater switching cost in RTs when switching away from a food task compared to switching towards a food task in negative emotion blocks, *F*(1,86) = 18.67, *p* < 0.001, η^2^_p_ = 0.178, but not in the neutral emotion blocks, *F*(1,86) = 1.20, *p* = 0.279, η^2^_p_ = 0.013. In contrast, the switching costs away and towards a food task were not different in healthy adolescents in the negative, *F*(1,86) = 1.39, *p* = 0.245, η^2^_p_ = 0.015, or neutral, *F*(1,86) = 1.19, *p* = 0.281, η^2^_p_ = 0.013, emotion blocks.

### 3.4 The Relationship Between Clinical Variables and Switching Costs Measures

Correlations between general switching costs in RTs and accuracy (i.e., averaged for emotion block type and switch type) and eating disorder symptoms severity (EDE‐Q global score as well as restraint, eating, weight, and shape concern subscales), self‐report cognitive flexibility (using the CFS) and other clinical variables (illness duration, %EBW) were assessed. The analyses were conducted separately for each group. The analyses did not reveal any significant correlations between eating disorder symptoms severity and switching cost measures in any of the groups (see Table 4). In contrast, higher levels of global eating disorder severity were associated with less subjective cognitive flexibility in both groups. Lastly, there were no significant correlations between switching cost measures and subjective cognitive flexibility among adolescents with AN (switching cost in RTs and CFS; *r* = −0.11, *p* = 0.47, switching cost in accuracy and CFS; *r* = 0.16, *p* = 0.29) nor among controls (switching cost in RTs and CFS; *r* = 0.12, *p* = 0.26, switching cost in accuracy and CFS; *r* = −0.01, *p* = 0.86).

**TABLE 4:** Correlations between clinical measures and general switching costs in response times (RTs) and accuracy.

	EDE‐Q global	EDE‐Q restraint subscale	EDE‐Q eating concern subscale	EDE‐Q weight concern subscale	EDE‐Q shape concern subscale
Adolescents with AN (*n* = 47)					
General switching cost in RTs	0.026	−0.000	0.091	−0.045	0.037
General switching cost in accuracy	−0.153	−0.161	0.003	−0.135	−0.222
CFS	−0.293*	−0.197	−0.343*	−0.224	−0.240
%EBW	−0.018	−0.031	−0.139	0.056	0.07
Illness duration^1^	−0.16	−0.19	−0.085	−0.064	−0.18
Controls (*n* = 41)					
General switching cost in RTs	−0.065	−0.08	−0.034	−0.084	−0.048
General switching cost in accuracy	0.069	0.042	0.08	0.09	0.051
CFS	−0.663***	−0.594***	−0.665***	−0.644***	−0.629***
%EBW	−0.109	−0.139	−0.144	−0.093	−0.055

*Note:* The analysis included all participants who filled in the questionnaires and completed the Emotion‐Food Task Switching Paradigm (*N* = 88).

Abbreviations: CFS, Cognitive Flexibility Scale; EDE‐Q, Eating Disorder Examination Questionnaire; %EBW, % expected body weight.

^1^ Illness duration was relevant just for adolescents with AN.

*p < 0.05, ***p < 0.001.

## 4 Discussion

The goal of the current study was to assess the influence of negative emotion on the ability to switch away or towards a food categorisation task among adolescents with AN. The results did not reveal a general deficit in set‐shifting abilities among adolescents with AN compared to healthy controls. However, the results did demonstrate aberrant patterns of set‐shifting among adolescents with AN as a function of exposure to disorder‐related state variables. Specifically, converging data from RTs and accuracy measures within the task demonstrated that adolescents with AN present reduced cost when switching from a non‐food task to a food‐task compared to switching from a food task to a non‐food task, but only in blocks that included exposure to negative emotional images. Switching costs when switching towards or away from a food task did not differ when emotionally neutral images were presented. Moreover, healthy adolescents did not present any differences between the switching cost types within the different emotion blocks. Planned comparisons revealed that the difference in switching costs among those with AN was driven by higher accuracy when switching towards a food task in negative compared to neutral emotion blocks, rather than lower accuracy in switching away from a food task under negative compared to neutral emotion blocks. In other words, while being exposed to negative emotional images, adolescents with AN disengaged more efficiently from a non‐food task and switched to a food task.

Previous studies suggested that individuals with AN exhibit an inflexible thinking style, which was proposed as one of the obstacles to successful treatment (Crane, Roberts, and Treasure 2007; Treasure and Schmidt 2013). It was theorized that set‐shifting difficulties drive inflexibility among individuals with AN (Smith et al. 2018). Although some evidence demonstrated set‐shifting difficulties within the framework of the task switching paradigm among adults with AN (Wu et al. 2014), the current study marks the first attempt to assess set‐shifting abilities in adolescents using the task switching paradigms while also manipulating emotional state and exposure to food stimuli.

The results of the current study demonstrate that when adolescents with AN engaged in a non‐food related task, namely, discriminating between frame colours, but then had to switch to a food task (i.e., sorting sweet and salty high‐calorie foods), they did this with greater efficiency compared to the opposite transition from a food task to a non‐food task. Importantly, this pattern occurred only when participants were exposed to negative images within the task, but not when neutral images were presented. The results imply that when adolescents with AN are in a negative emotional state, their engagement with food becomes more prominent, enabling them to transition quickly and accurately towards food‐related tasks. This notion converges with previous data showing that the experience of negative affect is positively associated with food preoccupation among those with AN (Seidel et al. 2016). The results of the current study extend this finding by showing that negative affect leads to faster and more accurate disengagement from a food‐unrelated task to a food task among adolescents with AN.

Previous findings demonstrated that a momentary experience of negative affect increases the propensity of individuals with AN to engage in disordered eating behaviours (Engel et al. 2016). The current study sheds light on the potential cognitive mechanism underlying this phenomenon by illustrating faster and more accurate switching towards engaging with food stimuli when negative emotion is induced. The central role played by negative affect in modulating eating‐related cognitions and behaviours corresponds with the models of AN as a predominant disorder of emotion dysregulation (Haynos and Fruzzetti 2011). The results of the current study support this notion by showing that negative affect plays an important role in altering cognitive functioning in a way that subserves disorder‐related behaviours (e.g., negative affect results in a faster and more efficient shift towards preoccupation with food among those with AN). Thus, promoting efficient coping with negative affect in treatment (e.g., by improving emotion regulation skills), may also reduce aberrant cognitive functioning. Indeed, previous studies have also shown that focussing on emotion processing and regulation improves outcomes in neuropsychological tests among individuals with AN to a greater extent than treatment as usual or cognitive training (Davies et al. 2012; Lock et al. 2018).

As stated above, the results of the study did not reveal a general deficit in set‐shifting abilities among adolescents with AN compared to healthy adolescents. The lack of group‐level differences in set‐shifting among adolescents with AN compared to controls converges with several previous studies that did not find set‐shifting difficulties among adolescents with AN compared to healthy controls using behavioural paradigms (Miles et al. 2020). Evidence regarding set‐shifting difficulties in adults with AN using such paradigms has been more consistent (see review in Miles et al. 2020). Given these data, suggestions that set‐shifting difficulties are a trait marker of AN (Holliday et al. 2005; King et al. 2019) seem less likely as evidence to support the presence of such difficulties at earlier stages of the disorder is weak. Longitudinal studies assessing the trajectory of set‐shifting abilities in AN are warranted to shed more light on this issue.

As opposed to behavioural paradigms assessing set‐shifting in individuals with AN, self‐report measures regarding cognitive inflexibility have been much more consistent. A recent systematic review (Miles et al. 2020) reported that both adults and adolescents with AN report marked difficulties in subjective measures of cognitive flexibility. The current study adds to these findings by revealing a robust effect size for reduced self‐report cognitive flexibility among adolescents with AN compared to healthy adolescents. Moreover, lower levels of self‐report cognitive flexibility were associated with greater global eating disorder symptom severity in both groups. Interestingly, this correlation was weaker in the AN group, likely due to the homogeneity within this sample, which resulted in limited variance in symptom severity and thus a potential range restriction effect. Behavioural measures of set shifting from the task switching paradigm did not correlate with the CFS nor with any of the eating disorder symptoms' severity measures in any of the groups. The absent relationship between objective and self‐report measures of clinical variables, as previously discussed (Enkavi and Poldrack 2021), suggests that the two types of measures might be tapping into distinct aspects of the studied variables. Moreover, recent data suggest that cognitive flexibility measures in restrictive AN are more closely associated with general psychopathology symptoms than eating disorder severity measures (Miranda‐Olivos et al. 2021). Given that our sample was mostly comprised of adolescents with restrictive AN, this could potentially account for the absent correlation between the task measures and eating disorder severity.

Several limitations of the current study should be acknowledged. First, the majority of patients in our study were diagnosed with the restrictive AN subtype and were all females, thus, limiting our ability to compare restrictive AN and binge eating/purging AN, or generalise the results to males with AN. Future studies should attempt to characterise set‐shifting abilities among males with AN, and assess potential differences in set‐shifting between AN subtypes as some studies have indicated that the two subtypes may differ in their neurocognitive profile (Wu et al. 2014). Additionally, the manipulation of emotion blocks within this study did not allow assessing potential differential influence of specific emotions (e.g., disgust, sadness, or anger) on set‐shifting abilities. It could be that some emotions that are more salient in patients with AN (e.g., disgust; Aharoni and Hertz 2012) may have a stronger impact on neurocognitive functions. Furthermore, despite differential results as a function of negative versus neutral emotion blocks, we did not verify participants self‐report mood ratings within the task. This was in order to mitigate potential risks of experimenter bias at the cost of the ability to verify that participants felt greater levels of negative emotionality after negative emotion blocks. Moreover, most of the participants with AN were partially weight restored and not in the acute phase of the disorder. However, previous studies did not find any difference in set‐shifting between adolescents with AN in acute versus weight‐restored condition of the disorder (Miles et al. 2020). Furthermore, in this study no associations between EBW% and switching costs were found. Another limitation of the study is that we did not control for hunger level among healthy adolescents. This may have increased uncontrolled variability in performance within this group as hunger can modulate attentional bias to food (see review in Hardman et al. 2021). The patient group performed the task between supervised meals within the inpatient unit and thus were not under a state of starvation. Finally, we did not measure the socioeconomic status of the adolescents.

Overall, the current study suggests that set‐shifting abilities among adolescents with AN are mostly intact. However, aberrant patterns in set‐shifting may arise as a result of environmental factors such as affective state and exposure to disorder‐related stimuli. The main finding we report is more efficient switching towards a food task when adolescents with AN are exposed to negative affective state. The study highlights the importance of considering potential interactions between cognitive abilities and environmental or disorder‐related situational factors in psychopathology research. Such research could potentially provide a more holistic and ecological overview of cognitive performance in different psychological disorders.

## Conflicts of Interest

The authors declare no conflicts of interest.

## Data Availability

The data that support the findings of this study are openly available in OSF at https://osf.io/h6wuy/?view_only=a61adaa2488648ed91e7687c358df981.
